# Effects of Movement Behaviors on Overall Health and Appetite Control: Current Evidence and Perspectives in Children and Adolescents

**DOI:** 10.1007/s13679-021-00467-5

**Published:** 2022-01-12

**Authors:** Valérie Julian, Ferdinand Haschke, Nicole Fearnbach, Julian Gomahr, Thomas Pixner, Dieter Furthner, Daniel Weghuber, David Thivel

**Affiliations:** 1 Department of Sport Medicine and Functional Explorations, University Teaching Hospital of Clermont-Ferrand, Diet and Musculoskeletal Health Team, Research Center in Human Nutrition, INRA, University of Clermont Auvergne, Clermont-Ferrand, France; 2 Obesity Research Unit, Paracelsus Medical University, Salzburg, Austria; 3 Department of Pediatrics, Paracelsus Medical University, Salzburg, Austria; 4 Pediatric Energy Balance Laboratory, Clinical Sciences Division, Pennington Biomedical Research Center, Baton Rouge, LA USA; 5 Department of Pediatrics and Adolescent Medicine, Salzkammergut-Klinikum, Vöcklabruck, Austria; 6 Laboratory AME2P, Research Center in Human Nutrition, University of Clermont Auvergne, Aubière, France

**Keywords:** Physical activity, Sedentary behaviors, Sleep, Energy intake, Appetite control, Pediatric obesity

## Abstract

***Purpose of Review*:**

To present the definitions and recommendations for movement behaviors in children and adolescents, including physical activity (PA), sedentary behaviors (SB), and sleep, and to provide an overview regarding their impact on health and obesity outcomes from childhood to adulthood, as well as interactions with appetite control.

***Recent Findings*:**

PA represents a variable proportion of daily energy expenditure and one can be active with high SB or vice versa. Studies have described movements across the whole day on a continuum from sleep to SB to varying intensities of PA. More PA, less SB (e.g., less screen time) and longer sleep are positively associated with indicators of physical health (e.g., lower BMI, adiposity, cardiometabolic risk) and cognitive development (e.g., motor skills, academic achievement). However, less than 10% of children currently meet recommendations for all three movement behaviors. Movement behaviors, adiposity, and related cardiometabolic diseases in childhood track into adolescence and adulthood. Furthermore, low PA/high SB profiles are associated with increased energy intake. Recent studies investigating energy balance regulation showed that desirable movement behavior profiles are associated with better appetite control and improved eating habits.

***Summary*:**

Early identification of behavioral phenotypes and a comprehensive approach addressing all key behaviors that directly affect energy balance will allow for individual strategies to prevent or treat obesity and its comorbidities. Investigating exercise as a potential “corrector” of impaired appetite control offers a promising weight management approach.

## Introduction

While the prevalence of childhood obesity seems to be stabilizing at a high level in many high-income countries, it continues to increase worldwide [1]. Obesity is the product of complex interactions between biological, genetic, environmental, and behavioral factors affecting energy intake relative to energy expenditure (energy balance) [2]. Both the quantity (number of calories consumed) and quality (ultra-processed foods, artificial sweeteners, etc.) of food and the amount of time spent in physical activity (PA) have been shown to be premonitory of subsequent weight gain [2, 3, 4, 5]. It has long been recognized that childhood obesity tracks across the life course. Whitaker et al. showed 20 years ago that obesity in childhood was a predictor of obesity in adulthood, regardless of whether the parents had obesity [6]. The likelihood of a child with obesity becoming an adult with obesity increases with the age of the child, with odds ratio for obesity in adulthood of 4.7, 8.8, and 22.3 for children having obesity between 3 and 5, 6 and 9, and 10 and 14 years of age, respectively [6]. These data suggest that earlier interventions are more likely to be effective [6], which is also supported by the results of interventional studies [7, 8].

Geserick et al. recently conducted both retrospective and prospective analyses of the course of BMI over time, emphasizing the association between BMI in early childhood and BMI in adolescence [9••]. Approximately half of adolescents with obesity had overweight or obesity from 5 years of age onward, and almost 90% of children suffering from obesity at 3 years of age suffered from overweight or obesity at adolescence [9••]. Among adolescents with obesity, the highest acceleration in annual BMI increments occurred during the preschool years, with a further rise in BMI percentile thereafter [9••].

Childhood obesity is associated with many adverse health outcomes, including cardiovascular (e.g. hypertension) and metabolic (e.g. insulin resistance, type 2 diabetes mellitus, dyslipidemia) disease-related comorbidities, the metabolic syndrome, and non-alcoholic fatty liver disease [2, 10, 11]. It has been recognized that childhood cardiovascular and metabolic diseases track into adulthood but also that excess adiposity in childhood constitutes a risk factor for the subsequent development of these diseases in adulthood, independently of the persistence of obesity in adulthood [12, 13]. A systematic review and meta-analysis showed that overweight or obesity in children from birth to 6 years of age was associated with an increased risk of metabolic syndrome in adulthood [12]. Twig et al. showed that adolescents with obesity had an increased risk of cardiovascular death over a 40-year period, with hazard ratios of 4.9 for death from coronary heart disease, 2.6 for death from stroke, and 3.5 for death from total cardiovascular causes [13], constituting the need for early intervention in childhood obesity. However, several drugs for the management of obesity have shown limited effectiveness, significant side effects, or are still understudied in younger children and preadolescents [14, 15, 16], and neither dietary nor PA interventions alone have been proven effective [17••, 18]. The combination of enhanced PA and improved nutrition has emerged as the cornerstone of therapy [17••, 18, 19, 20].

The majority of PA studies have shown that increasing PA not only improved body composition [21, 22], cardiometabolic factors [21, 23, 24], cardiorespiratory fitness [25, 26, 27], quality of life [28], but also mental and cognitive development [29, 30, 31, 32, 33, 34, 35•]. PA is regarded as a powerful marker of metabolic and psycho-social health [31, 32, 33] in children and adolescents, as it improves the 3 dimensions of health (i.e. physical, mental, and social health) as defined by the WHO (https://www.who.int/about/who-we-are/constitutionef). The recognition of cardiorespiratory fitness as an independent protective factor against adverse effects of obesity, particularly metabolic and cardiovascular diseases but also all-cause mortalities [36, 37], has contributed to the development of PA recommendations, in combination with nutritional interventions, to improve health [38•]. Early, effective, and innovative multidisciplinary strategies targeting multiple behaviors are thus required.

Activity energy expenditure (AEE), which refers to all energy expended above resting energy expenditure and dietary induced thermogenesis, represents between 20% of total energy expenditure (TEE) at 1 year old to 35% in young adults [39]. PA is the largest component of AEE. Other, but smaller, components of AEE include the energy expended above resting in sedentary behavior (SB) and sleep. Recent studies have investigated movements across the whole day on a continuum from sleep to SB to varying intensities of PA [40]. As noted below, there are positive associations of PA and sleep with health and negative associations of time spent in SB with health. PA not only affects energy expenditure but has also been shown to impact eating habits, food consumption, and appetite control [41•, 42•, 43•, 44]. Recently, the European Society for Pediatric Gastroenterology, Hepatology and Nutrition published an interesting review presenting the role of dietary factors, food habits, and lifestyle on the development of pediatric obesity, but did not fully address the importance of PA and SB [45]. To fill this gap, the present narrative review proposes a specific focus on movement behaviors, the available evidence linking these behaviors to overall health, how movement behaviors are associated with eating patterns/appetite control, and the potential mechanisms elucidated to date in children and adolescents with obesity.

## Behavior Patterns and Recommendations

### Physical Activities

PA is defined as any bodily movement produced by skeletal muscles resulting in a rise in energy expenditure (above resting energy expenditure). It can be categorized in daily life into occupational, sport, conditioning, household or other activities [46] and classified based on its intensity (metabolic equivalents of the task, METS), as light PA (LPA, 1.5–3 METS), moderate PA (MPA, 3–6 METS) and vigorous PA (6–9 METS), with the last two categories being often pooled under the term moderate to vigorous PA (MVPA) [47]. Exercise refers to a subset of PA planned with the objective to maintain or improve physical fitness, which is partly determined by PA patterns over weeks or months [48, 49, 50]. Health-related physical fitness (HRPF) includes cardiovascular endurance, muscle strength, flexibility, coordination, body composition, and metabolic components [48, 49, 50]. Both PA and HRPF in youth crucially depend on childhood motor skill development [51, 52].

### Sedentary Behaviors

While public health actors and strategies have been concentrated on promoting and evaluating PA, particularly leisure-time PA, studies in the last 15 years have also considered and assessed the physiology of children’s SB (as an equal partner to exercise physiology) and defined specific terminology [53, 54]. While SB refers to any waking behavior characterized by an energy expenditure below 1.5 METS (i.e. in a sitting, reclining or lying posture), physical inactivity is defined as an insufficient activity level to meet PA recommendations (described below) [55]. There is evidence to show that one can be very active while also engaging in high amounts of sitting time throughout the day. However, physical inactivity and SB health-related outcomes are commonly studied in isolation from each other, with additional focus on recreational screen time, which refers to the time spent in screen behaviors apart from school (i.e., watching TV, using a smartphone, tablet, computer) [56].

### 24-Hour Movement Behavior and Sleep

The most recent investigations have sought to capture children’s “24-h movement behavior” (i.e., pattern of movement across the whole day), with the objective to assess movements on a continuum from sleep to SB to MVPA [40]. Insufficient sleep has been shown to have negative impacts on children’s health [57]. Several explanations have been proposed, such as the activation of hormonal responses leading to an increase in appetite and food intake, the activation of inflammatory pathways or the reduction of free living energy expenditure as a result of reduced PA due to increased fatigue [57, 58]. Insufficient sleep has moreover been implicated in the relationship between high screen time and adverse health outcomes in pre-adolescents [59]. Children’s 24-h-movement behaviors, combining measures of PA, SB, and sleep, have thus garnered increased interest in both public health research and clinical practice, allowing scientists and practitioners to disentangle the independent and combined effects [41•, 60, 61, 62•, 63, 64, 65, 66].

### Current Recommendations

To optimize implementation into daily life, the current international recommendations simultaneously target PA, SB, and sleep. For younger children from 1 to 5 years of age, the recommendations are for at least 3 h daily PA, divided in short bouts of 10–20 min of active play spread throughout the day. Bouts should take the form of supervised games promoting reaching, stretching, crawling, running, kicking, throwing and catching, in order to acquire balance and motor skills, build strong bones and muscles, improve cardiorespiratory capacities, help achieve and maintain a healthy weight, and encourage self-confidence and independence [67•, 68]. For children from 5 to 12 years of age, the recommendations are for at least 60 min per day of MVPA, incorporating high impact activities (i.e. skipping, jumping, running or dancing) at least 3 days per week to promote bone health [61]. Recent recommendations have specified that children can accumulate PA through an average of 60 min of MVPA per day (not necessarily over the 7 days of the week) and should break up long periods of sitting as often as possible [38•]. Considering SB, recommendations advise to minimize sedentary time with a focus on reducing screen time, to be less than 1 h per day for children younger than 5 years old, and 2 h for older children [35•, 62•, 63]. The recommended sleep duration is between 10 and 13 h per day of uninterrupted, good quality sleep for children younger than 5 years old and between 9 and 11 h per day for older children [38•, 67•, 68]. The European Childhood Obesity Group (ECOG) has detailed these recommendations and specified its interests in pediatric obesity management [69].

## Current Evidence Linking Behaviors to Overall Health

### Relationship Between PA, SB and Health Outcomes

In the last three decades, many studies have demonstrated that PA positively influences motor skill development [70, 71], muscle strength and flexibility [72], bone mass accrual [73], cardiorespiratory fitness [25, 26, 27], body composition [21, 22], cardiometabolic factors (blood pressure, triglycerides, HDL-c, insulin-resistance, and lipoprotein levels) [21, 23, 24, 74], mental health [29, 31, 32, 34], quality of life [28], and cognitive development starting in early childhood [30, 31, 35•]. Thus, PA has been recognized as a powerful marker of metabolic and psychosocial health risks [75, 76, 77, 78]. More recently, a body of evidence has also shown that decreasing SB is associated with physical (less adiposity, lower waist circumference, better HRPF and lower metabolic and cardiovascular risk factors) and psychosocial health (greater pro-social behavioral, academic achievement) [35•, 60, 79]. As described above, desirable 24-h movement behavior patterns are beneficially associated with major health indicators in children, including BMI [62•, 63], adiposity [62•, 63], cardiometabolic health [63], HRPF [62•, 63], motor development and skills [62•, 63], quality of life [63, 64], cognition and academic achievement [62•, 63, 65]. Within the category of SB, recreational screen time has a specific strong association with adverse health outcomes, independently of the amount of PA [74, 80, 81]. Importantly, a dose–response relationship between PA, SB, and health indicators has been highlighted (i.e., the higher the frequency of PA and the less time spent sedentary, the greater the health benefit) [78, 82, 83, 84•].

Regarding pediatric overweight and obesity, evidence has shown strong inverse relationships between daily PA and BMI [85, 86, 87], body fat [88, 89], obesity-related metabolic diseases (insulin resistance, dyslipidemia, high blood pressure, and the metabolic syndrome) [35•, 79, 90•], and nonalcoholic fatty liver disease (NAFLD) [11, 91, 92]. In a multinational sample of 9-to-11–year-old children (n = 6539), low MVPA and high sedentary time were associated with higher odds of obesity (OR 0.49; 95% CI, 0.44–0.55 and OR 1.19; 95% CI, 1.08–1.30, respectively) [93]. This has also been confirmed for the combination of low MVPA and high screen time profile (OR, 1.71, 95CI 1.26–2.32) [80]. In the ISCOLE study, children meeting the three recommendations for PA, SB, and sleep were 70% less likely to suffer from obesity compared to those meeting none of the recommendations (OR 0.28, 95CI 0.18–0.45) [66]. In a meta-analysis carried out on more than 6000 children and adolescents from the International Children’s Accelerometry Database, crude models indicated that a 10-min increase in MVPA was inversely associated with the metabolic syndrome (OR 0.88, 95% CI 0.82–0.94), and that a 1-h increase in sedentary time was positively associated with the metabolic syndrome (OR 1.28, 95% CI 1.13–1.45) [90•]. Importantly, while PA, mainly through improved fitness, can improve cardiovascular and metabolic comorbidities independently of adiposity [94], we are still missing evidence regarding these relationships depending on SB and sleep, and the respect of the 24-h guidelines, altogether or separately.

### The Present Situation

**Fig. 1:**
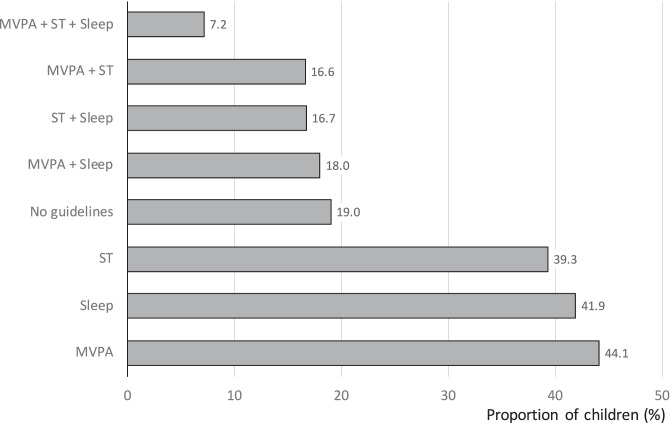
Proportion (%) of children meeting the moderate to vigorous physical activity (MVPA), sleep duration, screen time (ST) guidelines, combinations of these recommendations, and no guideline according to the International Study of Childhood Obesity, Lifestyle and the Environment (ISCOLE) [66]

The International Study of Childhood Obesity, Lifestyle and the Environment (ISCOLE), assessed 24-h movement patterns of more than 6000 preadolescents from socio-culturally and economically diverse countries worldwide (n = 12) [66]. The proportions of children meeting the MVPA, sleep duration, screen time guidelines, combinations of these recommendations, and no guideline, according to the ISCOLE, are summarize in Fig. 1. In the most recent decade, European and American epidemiologic studies using both objectively (accelerometry) and subjectively (self- or parent-report) assessed PA showed, that the percentage of children meeting the recommendation for MVPA declined with increasing age and that less than one-third of adolescents meet the recommendation for MVPA, which represents a mean daily percentage of awake time below 5% [95, 96, 97, 98]. Boys accumulate on average 10 to 15 more minutes of MVPA per day than girls [93]. Moreover, SB increases with age from 6 to almost 9 h per day at age 6 and 14, respectively [95, 99]. The UK, which is one of the first countries that proposed recommendations aiming at reducing SB, reports the higher sedentary time, with children aged 8–10 years old spend 80% of their waking hours in SB [98]. In Canadian children and adolescents, the mean amount of time spent in SB is 8.6 h (62% of the waking hours) and less than half of them meet guidelines for screen time [96]. Screen time, which represents about one-third of SB time, has increased worldwide at all ages, as toddlers, preschoolers, and children are now growing up in environments saturated with a variety of technologies [100], which are common correlates of SB and screen time [81]. Moreover, children and adolescents with reduced sleep time principally use extra-waking hours by spending more time in sedentary bouts [101•].

Children and adolescents with overweight or obesity spend even less time than their healthy-weight peers in MVPA [102, 103]. As overweight/obesity and low socioeconomic status coexist in developed countries, it is of interest that children from low socioeconomic backgrounds are at greater risk for high screen time and physical inactivity [104]. Otherwise, studies have largely shown that pre-adolescents accumulate less MVPA than younger children [96, 97]. The relative decline in MVPA affects both sexes from an early age; however, it is more pronounced in girls. A recent meta-analysis aiming to determine and compare the year-to-year changes in MVPA among children and adolescents showed an overall average decline of 3.4% (95% CI, − 5.9 to − 0.9) in boys and 5.3% (95% CI, − 7.6 to − 3.1) in girls between the age of 3 and 16 years, with notable declines in MVPA at age 9 for both boys (− 7.8%, 95% CI − 11.2 to − 4.4) and girls (− 10.2%, 95% CI − 14.2 to − 6.3) [105••]. Furthermore, this decline in MVPA with age would be compensated by an increase in the time spent in SB, with this compensation increasing with age [95]. This transition time between childhood and adolescence, which is a common time for levels of MVPA to decline and for SB/screen time to increase [81, 96], supports the premise that the promotion of MVPA should start before adolescence. Similarly, a high proportion of young children (70% in the USA) have delays in fundamental motor skill development, which may prevent them from undertaking more advanced PA participation [52].

### From Childhood to Adulthood

Importantly, behavior associated with overweight and obesity tracks across the life span. As illustrated by Blair in a theoretical model 30 years ago [106], childhood PA level, which has direct effects on child health, is a predictor of adolescent and adult PA level, and is therefore important for later health and obesity prevention during adolescence and adulthood [107, 108, 109, 110, 111, 112, 113]. Interestingly, a systematic review tracking both PA and SB from early to middle childhood revealed that SB tracks even more consistently over time than PA (83% of the included studies found moderate or large tracking versus 64% for PA) [114]. Furthermore, children who do not meet SB guidelines at the age of 10 would be 3 to 5 times more likely not to meet guidelines in adulthood [115]. The scientific evidence on relationships between childhood and adulthood behaviors and health has recently been actualized, illustrating that both childhood PA and SB have an impact on child health but also influences PA and SB later in life [116•], suggesting potential vicious cycles of worsening health with low PA/high SB profiles. For example, it has been suggested that compromised motor abilities in childhood could contribute to a low PA and high SB profile that would represent an important factor driving the effects of overweight, physical inactivity as low education achievement and low social insertion in late adolescence [117]. Similarly, BMI at the age of 10 has been negatively correlated with SB and screen time in adulthood, contributing to a less desirable PA/SB profile in middle-age [118]. Although longitudinal follow-up of sleep patterns between childhood and adulthood are rare in the literature, sleep duration at adolescence would be positively associated with sleep duration in young adults, and instances of self-report shorter sleep in mid-adulthood might appear to be formed in late adolescence [119].

Earlier intervention, particularly intervention beginning prior to puberty but with additional benefits of starting earlier in pre-puberty, seems to be more effective in sustaining reduced excess body fat over time [7, 8, 17••]. As the combination of nutrition and PA is necessary for sustained effects [17••, 120], a growing number of studies have investigated the impact of exercise on both energy expenditure (determined by daily movement behaviors, i.e., PA and SB) and energy intake. Although many studies have considered energy expenditure and energy intake separately, more recent work has begun to uncover interactions between activity levels and eating behaviors.

## Interaction Between Movement Behaviors, Eating Habits and Appetite Control

### Movement Behaviors Profiles and Eating Patterns

Recent work has focused on a comprehensive understanding of the relationships between all key behaviors — low PA, high SB, and unhealthy eating habits — that directly affect energy balance and favor weight gain. Both low levels of PA and high levels of SB have been associated with increased food intake and poor diet quality in children [121, 122]. Recently, Manz et al. showed in 9842 children and adolescents between 6 and 17 years of age that a higher PA level was associated with a higher intake of healthy food (i.e., fruits and vegetables) and a lower intake of unhealthy food (i.e., soft drinks, savory snacks) [42•]. Specifically, children with a high level of PA (1 h per day for 6–7 days per week) were more likely to consume a high amount of fruits (OR 2.0, 95% CI 1.6–2.7) and vegetables (OR 1.5, 95% CI 1.2–2) [42•]. These results are in line with other studies in school-aged children [123, 124, 125] and the HELENA study (Healthy Lifestyle in Europe by Nutrition in Adolescence), which also noted positive associations between PA level and healthy eating behaviors [126, 127•]. Moreover, the ISCOLE study, conducted in 5873 children aged 9 to 11 years old, showed that meeting screen time recommendations were strongly associated with more favorable eating behaviors [41•]. This is concordant with previous results suggesting that the two main mechanisms explaining the strong relationship between screen time and adiposity would be through insufficient sleep (i.e., blue light of screens that disrupts sleep patterns) and increased food intake (e.g., in front of the TV through distraction) [59, 80]. In younger children aged 5 years old from the EDEN mother–child cohort, the cluster of girls defined by high screen time exposure and unfavorable mealtime habits had the highest body fat percentages [128•]. Furthermore, this association evolved over time from ages 2 to 5 years [128•].

**Fig. 2:**
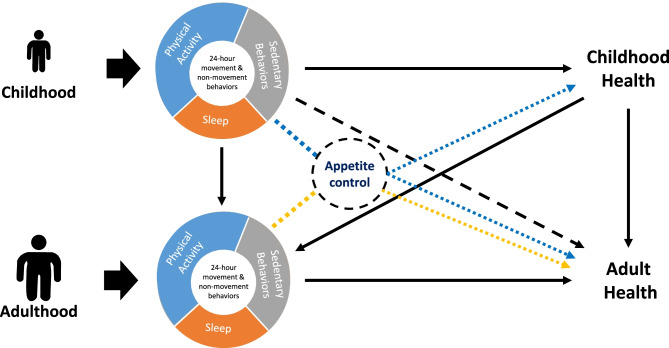
Relationships between 24-h movement behaviors, appetite control, and health from childhood to adulthood

From a physiological point of view, it has been demonstrated that energy intake follows a J-shaped curve across PA levels [129]. As a consequence, energy intake is in coherent homeostasis with energy expenditure only under conditions of high energy expenditure (i.e., the “linear” or “regulated zone”), but in low energy expenditure conditions (i.e., “non-linear” or “unregulated zone”), hedonic processes prevail over homeostatic regulating factors, leading to overconsumption [129, 130]. Evidence is presently accumulating to support the view that PA and SB can interact with appetite control and affect food intake in children with obesity [43•]. The relationships between 24-h movement behaviors, appetite control, and health from childhood to adulthood are illustrated in Fig. 2. Although this is a theoretical model, there is enough evidence suggesting an association between childhood and adulthood PA and eating behaviors and it can be advanced that improving appetite control and eating habits through exercise in children is highly likely to have beneficial effects life-long [131•].

### Exercise and Appetite Control: Impact of Exercise Intensity and Timing

Studies of PA focus on the selection of the best and most adapted exercise intensity and timing in order to optimize the effects on appetite control and on weight management. While studies have addressed the effects of exercise duration, modality, or induced-expenditure [121, 132, 133, 134, 135], intensity seems to be the primary exercise characteristic involved in the subsequent modulation of energy intake and appetite in youth [136]. The anorexigenic effect of an acute intense exercise (intensity above 65–70% of the individual maximal aerobic capacity) on subsequent food intake in children and adolescents with overweight/obesity is now clearly established [43•, 136]. Intensive exercise would thus act as a potential “corrector” of the impaired appetite control observed in youth with obesity, to help achieve homeostatic intake at the subsequent meal [43•].

The timing of exercise, including placement during the day (morning vs. afternoon or evening), the order/position (pre vs. post-meal), and the delay between exercise and meals, has recently appeared as another important parameter to consider [137•, 138]. Although the literature remains limited to date regarding the effect of exercising immediately before (pre-meal exercise) versus after a meal (post-meal exercise) in pediatric populations, Fillon et al. recently found reduced hunger feelings in pre-meal exercise conditions in adolescents with obesity [139]. On the other hand, in a well-controlled free-living study on primary school children, Mathieu et al. failed to observe any significant differences in energy intake between pre-meal exercise and post-meal exercise sessions [140]. To our knowledge, no study has addressed the effect of chronic exercise timing relative to meals on energy intake in children or adolescents. Most of the available evidence from acute studies suggests that exercising proximal to a meal might reduce energy intake or help youth avoid overconsumption [141, 142]. Studies in adolescents with both healthy weight [141] and obesity [142] have shown a reduction in energy intake after an acute exercise bout occurring 30–60 min before lunch (compared with 165–180 min before), which was not accompanied by a modification of appetite feelings nor compensation at the following meals. These findings suggest that there are no detrimental effects of pre-meal exercise in terms of hunger or frustration.

## Conclusions, Research Gaps and Recommendations for Routine Clinical Practice

This review presents the definitions and current recommendations for movement behaviors including PA, SB, and sleep, in children and adolescents. We also summarize current evidence on the negative impact of non-desirable childhood movement behavior patterns tracking into adolescence and adulthood (less PA/more SB (e.g. longer screen time)/shorter sleep) on major health outcomes (BMI, adiposity, cardiometabolic risk, motors/skills development, cardiorespiratory fitness, quality of life, cognition and education achievement) in childhood, which track into adolescence and adulthood. Scientific evidence shows associations between low PA/high SB profiles and increased food intake and poor diet quality in children. Recent studies have focused on the complex interactions between all these key behaviors that directly affect energy balance and weight gain. Studies are identifying exercise as a potential “corrector” of impaired appetite control in youth with obesity, which offers a promising weight management approach. However, there are still substantial research gaps and recommendations for future research, as detailed in Tables 1 and 2. Since not every child enjoys, or has access to opportunities for, engaging in the same PA, schools and health care providers, might consider emphasizing a precision medicine approach to encouraging PA based on community-based opportunities and individualized preferences.

**Table 1:** Research gaps

• More studies should focus on preschool children’s behaviors (as a fundamental phase for long term obesity management), including the family context/situation, with longer follow-up periods to evaluate prospectively the impact of behavioral phenotypes and interventions that have sustained effects
• As “a shift in focus away from individual behaviors toward the wider environment” has recently been required [131•], targeting the environment children grow up and thus providing the prerequisites for them to develop optimal movement behaviors is warranted
• There is still insufficient evidence available to fully describe the dose–response relationships (as the threshold values) between PA and obesity-related health outcomes, and whether the associations vary by the “type” (i.e., aerobic vs. strength exercise) or the “domain” (active transport such as walking and cycling vs. physical education vs. sports/recreation) of PA
• More trials are needed to investigate the timing of exercise (proximity to meals and the effect of morning vs. afternoon exercises) in children and adolescents, as an approach to moderate energy balance. These would be especially relevant in free-living, school-based settings to optimize public health strategies
• As studies present a high level of methodological heterogeneity, more consistent and standardized methods are needed when investigating PA (timing, intensity, duration, modality), energy and macronutrient intakes (objective measurements), and food preference in children with obesity

**Table 2:** Recommendations for routine clinical practice

• We recommend to follow international movement recommendations for each age group that simultaneously target PA, SB (including screen time), and sleep (chapter 2, current recommendations), adapting them to individual circumstances and capacity
• Additional PA beyond 60 min of daily MVPA appears to be better for various health outcomes. There are dose-responses relationship between PA, sedentary time (including recreational screen time) and health outcomes
• More attention should be given starting in early childhood and particularly for children from low familial socioeconomic or educational status, who are more at risk for both physical inactivity and high screen time
• Children with overweight or obesity should receive an individual behavioral assessment and tailored support, targeting concomitant modification of key behaviors while taking into account circumstances and capacities, such as familial environment and education
• More attention should be given to timing of energy balance-related behaviors, particularly in school environments, as intense exercising (above 65–70% of the individual’s maximal aerobic capacities) during the morning and proximal to a meal might improve appetite control and reduce energy intake

## Data Availability

Not applicable.

## References

[1] NCD Risk Factor Collaboration (NCD-RisC). Worldwide trends in body-mass index, underweight, overweight, and obesity from (1975). to 2016: a pooled analysis of 2416 population-based measurement studies in 128·9 million children, adolescents, and adults. Lancet.

[2] Kansra AR, Lakkunarajah S, Jay MS. Childhood and adolescent obesity: a review. Front Pediatr. 2020;8:581461. 10.3389/fped.2020.581461 PMC7835259 33511092

[3] Sirkka O, Fleischmann M, Abrahamse-Berkeveld M, Halberstadt J, Olthof MR, Seidell JC (2021). Dietary patterns in early childhood and the risk of childhood overweight: the GECKO Drenthe Birth Cohort. Nutrients.

[4] Chang K, Khandpur N, Neri D, Touvier M, Huybrechts I, Millett C, et al. Association between childhood consumption of ultraprocessed food and adiposity trajectories in the Avon longitudinal study of parents and children birth cohort. JAMA Pediatr. 2021;175:e211573. 10.1001/jamapediatrics.2021.1573 PMC8424476 34125152

[5] Hu FB, Malik VS (2010). Sugar-sweetened beverages and risk of obesity and type 2 diabetes: epidemiologic evidence. Physiol Behav.

[6] Whitaker RC, Wright JA, Pepe MS, Seidel KD, Dietz WH (1997). Predicting obesity in young adulthood from childhood and parental obesity. N Engl J Med.

[7] Reinehr T, Kleber M, Lass N, Toschke AM (2010). Body mass index patterns over 5 y in obese children motivated to participate in a 1-y lifestyle intervention: age as a predictor of long-term success. Am J Clin Nutr.

[8] Magarey AM, Perry RA, Baur LA, Steinbeck KS, Sawyer M, Hills AP (2011). A parent-led family-focused treatment program for overweight children aged 5 to 9 years: the PEACH RCT. Pediatrics.

[9] Geserick M, Vogel M, Gausche R, Lipek T, Spielau U, Keller E (2018). Acceleration of BMI in early childhood and risk of sustained obesity. N Engl J Med.

[10] GBD 2015 Obesity Collaborators, Afshin A, Forouzanfar MH, Reitsma MB, Sur P, Estep K, et al. Health effects of overweight and obesity in 195 countries over 25 years. N Engl J Med. 2017;377:13–27. 10.1056/NEJMoa1614362 PMC5477817 28604169

[11] Temple JL, Cordero P, Li J, Nguyen V, Oben JA (2016). A guide to non-alcoholic fatty liver disease in childhood and adolescence. Int J Mol Sci.

[12] Kim J, Lee I, Lim S (2017). Overweight or obesity in children aged 0 to 6 and the risk of adult metabolic syndrome: a systematic review and meta-analysis. J Clin Nurs.

[13] Twig G, Yaniv G, Levine H, Leiba A, Goldberger N, Derazne E, et al. Body-mass index in 2.3 million adolescents and cardiovascular death in adulthood. N Engl J Med. 2016;374:2430–40. 10.1056/NEJMoa1503840 27074389

[14] Mead E, Atkinson G, Richter B, Metzendorf M-I, Baur L, Finer N, et al. Drug interventions for the treatment of obesity in children and adolescents. Cochrane Database Syst Rev. 2016;11:CD012436. 10.1002/14651858.CD012436 PMC6472619 27899001

[15] Grandone A, Di Sessa A, Umano GR, Toraldo R, Miraglia Del Giudice E (2018). New treatment modalities for obesity. Best Pract Res Clin Endocrinol Metab.

[16] Singhal V, Sella AC, Malhotra S (2021). Pharmacotherapy in pediatric obesity: current evidence and landscape. Curr Opin Endocrinol Diabetes Obes.

[17] •• Brown T, Moore TH, Hooper L, Gao Y, Zayegh A, Ijaz S, et al. Interventions for preventing obesity in children. Cochrane Database of Systematic Reviews [Internet]. John Wiley & Sons, Ltd; 2019 [cited 2021 Jun 24]. Available from: https://www.cochranelibrary.com/cdsr/doi/10.1002/14651858.CD001871.pub4/full This Chochrane Database systematic review determines the effectiveness of a range of interventions that include diet or physical activity components, or both, designed to prevent obesity in children.

[18] Steinbeck KS, Lister NB, Gow ML, Baur LA (2018). Treatment of adolescent obesity. Nat Rev Endocrinol.

[19] Cardel MI, Atkinson MA, Taveras EM, Holm J-C, Kelly AS (2020). Obesity treatment among adolescents: a review of current evidence and future directions. JAMA Pediatr.

[20] Kumar S, Kelly AS (2017). Review of childhood obesity: from epidemiology, etiology, and comorbidities to clinical assessment and treatment. Mayo Clin Proc.

[21] Stoner L, Rowlands D, Morrison A, Credeur D, Hamlin M, Gaffney K (2016). Efficacy of exercise intervention for weight loss in overweight and obese adolescents: meta-analysis and implications. Sports Med.

[22] Waters E, de Silva-Sanigorski A, Hall BJ, Brown T, Campbell KJ, Gao Y, et al. Interventions for preventing obesity in children. Cochrane Database Syst Rev. 2011;CD001871. 10.1002/14651858.CD001871.pub3 22161367

[23] Cesa CC, Sbruzzi G, Ribeiro RA, Barbiero SM, de Oliveira PR, Eibel B (2014). Physical activity and cardiovascular risk factors in children: meta-analysis of randomized clinical trials. Prev Med.

[24] Marson EC, Delevatti RS, Prado AKG, Netto N, Kruel LFM (2016). Effects of aerobic, resistance, and combined exercise training on insulin resistance markers in overweight or obese children and adolescents: a systematic review and meta-analysis. Prev Med.

[25] Cao M, Quan M, Zhuang J (2019). Effect of high-intensity interval training versus moderate-intensity continuous training on cardiorespiratory fitness in children and adolescents: a meta-analysis. Int J Environ Res Public Health.

[26] Oliveira A, Monteiro Â, Jácome C, Afreixo V, Marques A (2017). Effects of group sports on health-related physical fitness of overweight youth: a systematic review and meta-analysis. Scand J Med Sci Sports.

[27] Szeszulski J, Lorenzo E, Shaibi GQ, Buman MP, Vega-López S, Hooker SP, et al. Effectiveness of early care and education center-based interventions for improving cardiovascular fitness in early childhood: a systematic review and meta-analysis. Prev Med Rep. 2019;15:100915. 10.1016/j.pmedr.2019.100915 PMC6598036 31297309

[28] Marker AM, Steele RG, Noser AE (2018). Physical activity and health-related quality of life in children and adolescents: a systematic review and meta-analysis. Health Psychol.

[29] Andermo S, Hallgren M, Nguyen TTD, Jonsson S, Petersen S, Friberg M, et al. School-related physical activity interventions and mental health among children: a systematic review and meta-analysis. Sports Med Open. 2020;6:25. 10.1186/s40798-020-00254-x PMC7297899 32548792

[30] Sun X, Li Y, Cai L, Wang Y (2021). Effects of physical activity interventions on cognitive performance of overweight or obese children and adolescents: a systematic review and meta-analysis. Pediatr Res.

[31] Sember V, Jurak G, Kovač M, Morrison SA, Starc G (2020). Children’s physical activity, academic performance, and cognitive functioning: a systematic review and meta-analysis. Front Public Health.

[32] Petty KH, Davis CL, Tkacz J, Young-Hyman D, Waller JL (2009). Exercise effects on depressive symptoms and self-worth in overweight children: a randomized controlled trial. J Pediatr Psychol.

[33] Tkacz J, Young-Hyman D, Boyle CA, Davis CL (2008). Aerobic exercise program reduces anger expression among overweight children. Pediatr Exerc Sci.

[34] Sund AM, Larsson B, Wichstrøm L (2011). Role of physical and sedentary activities in the development of depressive symptoms in early adolescence. Soc Psychiatry Psychiatr Epidemiol.

[35] Carson V, Tremblay MS, Chaput J-P, Chastin SFM (2016). Associations between sleep duration, sedentary time, physical activity, and health indicators among Canadian children and youth using compositional analyses. Appl Physiol Nutr Metab.

[36] Nyström CD, Henriksson P, Martínez-Vizcaíno V, Medrano M, Cadenas-Sanchez C, Arias-Palencia NM (2017). Does cardiorespiratory fitness attenuate the adverse effects of severe/morbid obesity on cardiometabolic risk and insulin resistance in children? A pooled analysis. Diabetes Care American Diabetes Association.

[37] Ruiz JR, Castro-Piñero J, Artero EG, Ortega FB, Sjöström M, Suni J (2009). Predictive validity of health-related fitness in youth: a systematic review. Br J Sports Med.

[38] Chaput J-P, Willumsen J, Bull F, Chou R, Ekelund U, Firth J (2020). 2020 WHO guidelines on physical activity and sedentary behaviour for children and adolescents aged 5–17 years: summary of the evidence. Int J Behav Nutr Phys Act.

[39] Westerterp KR (2017). Control of energy expenditure in humans. Eur J Clin Nutr.

[40] Tremblay MS, Carson V, Chaput J-P, Connor Gorber S, Dinh T, Duggan M (2016). Canadian 24-Hour Movement Guidelines for Children and Youth: An Integration of Physical Activity, Sedentary Behaviour, and Sleep. Appl Physiol Nutr Metab.

[41] Thivel D, Tremblay MS, Katzmarzyk PT, Fogelholm M, Hu G, Maher C (2019). Associations between meeting combinations of 24-hour movement recommendations and dietary patterns of children: a 12-country study. Prev Med.

[42] • Manz K, Mensink GBM, Finger JD, Haftenberger M, Brettschneider A-K, Lage Barbosa C, et al. Associations between physical activity and food intake among children and adolescents: results of KiGGS wave 2. Nutrients. 2019;11. This cross-sectional study analyses the association between food intake and physical activity among 9842 children and adolescents aged 6 to 17 years, from the German Health Interview and Examination Survey for Children and Adolescents (KiGGS Wave 2). 10.3390/nu11051060 PMC6566319 31083548

[43] Thivel D, Finlayson G, Blundell JE (2019). Homeostatic and neurocognitive control of energy intake in response to exercise in pediatric obesity: a psychobiological framework. Obes Rev.

[44] Fillon A, Beaulieu K, Miguet M, Bailly M, Finlayson G, Julian V, et al. Delayed meal timing after exercise is associated with reduced appetite and energy intake in adolescents with obesity. Pediatr Obes. 2020;15:e12651. 10.1111/ijpo.12651 32372568

[45] Verduci E, Bronsky J, Embleton N, Gerasimidis K, Indrio F, Köglmeier J (2021). Role of dietary factors, food habits, and lifestyle in childhood obesity development: a position paper from the European Society for Paediatric Gastroenterology, Hepatology and Nutrition Committee on Nutrition. J Pediatr Gastroenterol Nutr.

[46] Global Recommendations on Physical Activity for Health [Internet]. Geneva: World Health Organization; 2010 [cited 2021 Jun 25]. Available from: http://www.ncbi.nlm.nih.gov/books/NBK305057/26180873

[47] Sirard JR, Pate RR (2001). Physical activity assessment in children and adolescents. Sports Med.

[48] Pate RR, Dowda M, Ross JG (1990). Associations between physical activity and physical fitness in American children. Am J Dis Child.

[49] Katzmarzyk PT, Malina RM, Song TM, Bouchard C (1998). Physical activity and health-related fitness in youth: a multivariate analysis. Med Sci Sports Exerc.

[50] Huang Y-C, Malina RM (2002). Physical activity and health-related physical fitness in Taiwanese adolescents. J Physiol Anthropol Appl Human Sci.

[51] Slykerman S, Ridgers ND, Stevenson C, Barnett LM (2016). How important is young children’s actual and perceived movement skill competence to their physical activity?. J Sci Med Sport.

[52] Gu X, Tamplain PM, Chen W, Zhang T, Keller MJ, Wang J (2021). A mediation analysis of the association between fundamental motor skills and physical activity during middle childhood. Children (Basel).

[53] Tremblay MS, Aubert S, Barnes JD, Saunders TJ, Carson V, Latimer-Cheung AE (2017). Sedentary Behavior Research Network (SBRN) — terminology consensus project process and outcome. Int J Behav Nutr Phys Act.

[54] Hamilton MT, Hamilton DG, Zderic TW (2004). Exercise physiology versus inactivity physiology: an essential concept for understanding lipoprotein lipase regulation. Exerc Sport Sci Rev.

[55] Tremblay MS, LeBlanc AG, Kho ME, Saunders TJ, Larouche R, Colley RC (2011). Systematic review of sedentary behaviour and health indicators in school-aged children and youth. Int J Behav Nutr Phys Act.

[56] Chaput JP, Lambert M, Mathieu ME, Tremblay MS, Loughlin JO, Tremblay A. Physical activity vs. sedentary time: independent associations with adiposity in children. Pediatr Obes. 2012;7:251–8. 10.1111/j.2047-6310.2011.00028.x 22461356

[57] Cappuccio FP, Taggart FM, Kandala N-B, Currie A, Peile E, Stranges S (2008). Meta-analysis of short sleep duration and obesity in children and adults. Sleep.

[58] St-Onge M-P (2017). Sleep-obesity relation: underlying mechanisms and consequences for treatment. Obes Rev.

[59] LeBlanc AG, Gunnell KE, Prince SA, Saunders TJ, Barnes JD, Chaput J-P (2017). The ubiquity of the screen: an overview of the risks and benefits of screen time in our modern world. Translational Journal of the American College of Sports Medicine.

[60] Carson V, Hunter S, Kuzik N, Gray CE, Poitras VJ, Chaput J-P (2016). Systematic review of sedentary behaviour and health indicators in school-aged children and youth: an update. Appl Physiol Nutr Metab.

[61] Chaput J-P, Colley RC, Aubert S, Carson V, Janssen I, Roberts KC (2017). Proportion of preschool-aged children meeting the Canadian 24-Hour Movement Guidelines and associations with adiposity: results from the Canadian Health Measures Survey. BMC Public Health.

[62] Kuzik N, Poitras VJ, Tremblay MS, Lee E-Y, Hunter S, Carson V (2017). Systematic review of the relationships between combinations of movement behaviours and health indicators in the early years (0–4 years). BMC Public Health.

[63] Saunders TJ, Gray CE, Poitras VJ, Chaput J-P, Janssen I, Katzmarzyk PT (2016). Combinations of physical activity, sedentary behaviour and sleep: relationships with health indicators in school-aged children and youth. Appl Physiol Nutr Metab.

[64] Sampasa-Kanyinga H, Standage M, Tremblay MS, Katzmarzyk PT, Hu G, Kuriyan R (2017). Associations between meeting combinations of 24-h movement guidelines and health-related quality of life in children from 12 countries. Public Health.

[65] Lien A, Sampasa-Kanyinga H, Colman I, Hamilton HA, Chaput J-P (2020). Adherence to 24-hour movement guidelines and academic performance in adolescents. Public Health.

[66] Roman-Viñas B, Chaput J-P, Katzmarzyk PT, Fogelholm M, Lambert EV, Maher C (2016). Proportion of children meeting recommendations for 24-hour movement guidelines and associations with adiposity in a 12-country study. Int J Behav Nutr Phys Act.

[67] Tremblay MS, Chaput J-P, Adamo KB, Aubert S, Barnes JD, Choquette L (2017). Canadian 24-Hour Movement Guidelines for the early years (0–4 years): an integration of physical activity, sedentary behaviour, and sleep. BMC Public Health.

[68] Willumsen J, Bull F (2020). Development of WHO guidelines on physical activity, sedentary behavior, and sleep for children less than 5 years of age. J Phys Act Health.

[69] O’Malley G, Thivel D. Physical Activity And Play In Children Who Are Obese [Internet]. Childhood Obesity eBook. 2015 [cited 2021 Jun 25]. Available from: https://ebook.ecog-obesity.eu/chapter-energy-expenditure-physical-activity/physical-activity-play-children-obese/

[70] Lillegard WA, Brown EW, Wilson DJ, Henderson R, Lewis E (1997). Efficacy of strength training in prepubescent to early postpubescent males and females: effects of gender and maturity. Pediatr Rehabil.

[71] Serbescu C, Flora D, Hantiu I, Greene D, Laurent Benhamou C, Courteix D (2006). Effect of a six-month training programme on the physical capacities of Romanian schoolchildren. Acta Paediatr.

[72] Faigenbaum AD, Westcott WL, Loud RL, Long C. The effects of different resistance training protocols on muscular strength and endurance development in children. Pediatrics. 1999;104:e5. 10.1542/peds.104.1.e5 10390291

[73] Bailey DA, McKay HA, Mirwald RL, Crocker PR, Faulkner RA (1999). A six-year longitudinal study of the relationship of physical activity to bone mineral accrual in growing children: the university of Saskatchewan bone mineral accrual study. J Bone Miner Res.

[74] Chaput J-P, Saunders TJ, Mathieu M-È, Henderson M, Tremblay MS, O’Loughlin J (2013). Combined associations between moderate to vigorous physical activity and sedentary behaviour with cardiometabolic risk factors in children. Appl Physiol Nutr Metab.

[75] Kumar B, Robinson R, Till S (2015). Physical activity and health in adolescence. Clin Med (Lond).

[76] Poitras VJ, Gray CE, Borghese MM, Carson V, Chaput J-P, Janssen I (2016). Systematic review of the relationships between objectively measured physical activity and health indicators in school-aged children and youth. Appl Physiol Nutr Metab.

[77] Ortega FB, Ruiz JR, Castillo MJ, Sjöström M (2008). Physical fitness in childhood and adolescence: a powerful marker of health. Int J Obes (Lond).

[78] Janssen I, Leblanc AG (2010). Systematic review of the health benefits of physical activity and fitness in school-aged children and youth. Int J Behav Nutr Phys Act.

[79] Poitras VJ, Gray CE, Janssen X, Aubert S, Carson V, Faulkner G (2017). Systematic review of the relationships between sedentary behaviour and health indicators in the early years (0–4 years). BMC Public Health.

[80] Crowe M, Sampasa-Kanyinga H, Saunders TJ, Hamilton HA, Benchimol EI, Chaput J-P (2020). Combinations of physical activity and screen time recommendations and their association with overweight/obesity in adolescents. Can J Public Health.

[81] LeBlanc AG, Katzmarzyk PT, Barreira TV, Broyles ST, Chaput JP, Church TS, et al. Correlates of total sedentary time and screen time in 9–11 year-old children around the world: the international study of childhood obesity, lifestyle and the environment. PLoS One. 2015;10:e0129622. 10.1371/journal.pone.0129622 PMC4465981 26068231

[82] Wu XY, Han LH, Zhang JH, Luo S, Hu JW, Sun K. The influence of physical activity, sedentary behavior on health-related quality of life among the general population of children and adolescents: a systematic review. PLoS One. Public Library Sci. 2017;12:e0187668. 10.1371/journal.pone.0187668 PMC5679623 29121640

[83] Nevill AM, Duncan MJ, Sandercock G (2020). The dose–response association between V̇O2peak and self-reported physical activity in children. Journal of Sports Sciences Routledge.

[84] Sriram K, Mulder HS, Frank HR, Santanam TS, Skinner AC, Perrin EM (2021). The dose-response relationship between physical activity and cardiometabolic health in adolescents. Am J Prev Med.

[85] Klein-Platat C, Oujaa M, Wagner A, Haan MC, Arveiler D, Schlienger JL (2005). Physical activity is inversely related to waist circumference in 12-y-old French adolescents. Int J Obes (Lond).

[86] Ekelund U, Sardinha LB, Anderssen SA, Harro M, Franks PW, Brage S (2004). Associations between objectively assessed physical activity and indicators of body fatness in 9- to 10-y-old European children: a population-based study from 4 distinct regions in Europe (the European Youth Heart Study). Am J Clin Nutr.

[87] Page A, Cooper AR, Stamatakis E, Foster LJ, Crowne EC, Sabin M (2005). Physical activity patterns in nonobese and obese children assessed using minute-by-minute accelerometry. Int J Obes (Lond).

[88] Ball EJ, O’Connor J, Abbott R, Steinbeck KS, Davies PS, Wishart C (2001). Total energy expenditure, body fatness, and physical activity in children aged 6–9 y. Am J Clin Nutr.

[89] Rush EC, Plank LD, Davies PSW, Watson P, Wall CR (2003). Body composition and physical activity in New Zealand Maori, Pacific and European children aged 5–14 years. Br J Nutr.

[90] • Renninger M, Hansen BH, Steene-Johannessen J, Kriemler S, Froberg K, Northstone K, et al. Associations between accelerometry measured physical activity and sedentary time and the metabolic syndrome: a meta-analysis of more than 6000 children and adolescents. Pediatr Obes. 2020;15:e12578. This meta-analysis examines the cross-sectional associations between the metabolic syndrome and physical activity and sedentary time in 6009 children and adolescents from 8 studies of the International Children's Accelerometry Database. 10.1111/ijpo.12578 PMC7003500 31709781

[91] Anderson EL, Fraser A, Howe LD, Callaway MP, Sattar N, Day C (2016). Physical activity is prospectively associated with adolescent nonalcoholic fatty liver disease. J Pediatr Gastroenterol Nutr.

[92] Gibson PS, Lang S, Dhawan A, Fitzpatrick E, Blumfield ML, Truby H (2017). Systematic review: nutrition and physical activity in the management of paediatric nonalcoholic fatty liver disease. J Pediatr Gastroenterol Nutr.

[93] Katzmarzyk PT, Barreira TV, Broyles ST, Champagne CM, Chaput J-P, Fogelholm M (2015). Physical activity, sedentary time, and obesity in an international sample of children. Med Sci Sports Exerc.

[94] Sævarsson ES, Magnússon KT, Sveinsson T, Jóhannsson E, Arngrímsson SÁ (2016). The association of cardiorespiratory fitness to health independent of adiposity depends upon its expression. Ann Hum Biol.

[95] Husu P, Vähä-Ypyä H, Vasankari T (2016). Objectively measured sedentary behavior and physical activity of Finnish 7- to 14-year-old children- associations with perceived health status: a cross-sectional study. BMC Public Health.

[96] Colley RC, Garriguet D, Janssen I, Craig CL, Clarke J, Tremblay MS (2011). Physical activity of Canadian children and youth: accelerometer results from the 2007 to 2009 Canadian Health Measures Survey. Health Rep.

[97] Kalman M, Inchley J, Sigmundova D, Iannotti RJ, Tynjälä JA, Hamrik Z (2015). Secular trends in moderate-to-vigorous physical activity in 32 countries from 2002 to 2010: a cross-national perspective. Eur J Public Health.

[98] Pearce MS, Basterfield L, Mann KD, Parkinson KN, Adamson AJ, Reilly JJ, et al. Early predictors of objectively measured physical activity and sedentary behaviour in 8–10 year old children: the Gateshead Millennium Study. PLoS One. 2012;7:e37975. 10.1371/journal.pone.0037975 PMC3380043 22745660

[99] Matthews CE, Chen KY, Freedson PS, Buchowski MS, Beech BM, Pate RR (2008). Amount of time spent in sedentary behaviors in the United States, 2003–2004. Am J Epidemiol.

[100] Council on communications and media. Media and Young Minds. Pediatrics. 2016;138:e20162591. 10.1542/peds.2016-2591 27940793

[101] Gába A, Dygrýn J, Štefelová N, Rubín L, Hron K, Jakubec L (2020). How do short sleepers use extra waking hours? A compositional analysis of 24-h time-use patterns among children and adolescents. Int J Behav Nutr Phys Act.

[102] Trost SG, Kerr LM, Ward DS, Pate RR (2001). Physical activity and determinants of physical activity in obese and non-obese children. Int J Obes Relat Metab Disord.

[103] Elmesmari R, Martin A, Reilly JJ, Paton JY. Comparison of accelerometer measured levels of physical activity and sedentary time between obese and non-obese children and adolescents: a systematic review. BMC Pediatr [Internet]. 2018 [cited 2021 Jan 14];18. Available from: https://www.ncbi.nlm.nih.gov/pmc/articles/PMC5844092/10.1186/s12887-018-1031-0 PMC5844092 29523101

[104] Yang-Huang J, van Grieken A, Wang L, Jansen W, Raat H (2020). Clustering of sedentary behaviours, physical activity, and energy-dense food intake in six-year-old children: associations with family socioeconomic status. Nutrients.

[105] •• Farooq A, Martin A, Janssen X, Wilson MG, Gibson AM, Hughes A, et al. Longitudinal changes in moderate-to-vigorous-intensity physical activity in children and adolescents: a systematic review and meta-analysis. Obes Rev. 2020;21:e12953. This meta-analysis aims to determine and compare the year-to-year changes in MVPA among 22091 children and adolescents (aged 3 to 19 years old), with separated analysis for boys and girls. 10.1111/obr.12953 PMC6916562 31646739

[106] Blair SN, Clark DG, Cureton KJ, Powell KE. Exercise and fitness in childhood : implications for lifetime of health [Internet]. McGraw-Hill. New-York: McGrow-Hill; 1989 [cited 2021 Jun 25]. Available from: https://ci.nii.ac.jp/naid/20001520927/

[107] Boreham C, Riddoch C (2001). The physical activity, fitness and health of children. J Sports Sci.

[108] Parsons TJ, Power C, Logan S, Summerbell CD (1999). Childhood predictors of adult obesity: a systematic review. Int J Obes Relat Metab Disord.

[109] Kemper HC, Post GB, Twisk JW, van Mechelen W (1999). Lifestyle and obesity in adolescence and young adulthood: results from the Amsterdam Growth And Health Longitudinal Study (AGAHLS). Int J Obes Relat Metab Disord.

[110] Murphy MH, Rowe DA, Woods CB. Sports participation in youth as a predictor of physical activity: a 5-year longitudinal study. J Phys Act Health Hum Kinet Inc. 2016;13:704–11. 10.1123/jpah.2015-0526 26800567

[111] Yang X, Telama R, Leskinen E, Mansikkaniemi K, Viikari J, Raitakari OT (2007). Testing a model of physical activity and obesity tracking from youth to adulthood: the cardiovascular risk in young Finns study. Int J Obes.

[112] Bélanger M, Sabiston CM, Barnett TA, O’Loughlin E, Ward S, Contreras G (2015). Number of years of participation in some, but not all, types of physical activity during adolescence predicts level of physical activity in adulthood: results from a 13-year study. Int J Behav Nutr Phys Act.

[113] Schmidt MD, Magnussen CG, Rees E, Dwyer T, Venn AJ (2016). Childhood fitness reduces the long-term cardiometabolic risks associated with childhood obesity. Int J Obes (Lond).

[114] Jones RA, Hinkley T, Okely AD, Salmon J (2013). Tracking physical activity and sedentary behavior in childhood: a systematic review. American Journal of Preventive Medicine Elsevier.

[115] Busschaert C, Cardon G, Cauwenberg JV, Maes L, Damme JV, Hublet A (2015). Tracking and predictors of screen time from early adolescence to early adulthood: a 10-year follow-up study. Journal of Adolescent Health Elsevier.

[116] Thivel D, Chaput JP, Duclos M (2018). Integrating sedentary behavior in the theoretical model linking childhood to adulthood activity and health?. An updated framework Physiology & Behavior.

[117] Kantomaa MT, Stamatakis E, Kankaanpää A, Kaakinen M, Rodriguez A, Taanila A (2013). Physical activity and obesity mediate the association between childhood motor function and adolescents’ academic achievement. Proc Natl Acad Sci U S A.

[118] Smith L, Gardner B, Hamer M (2015). Childhood correlates of adult TV viewing time: a 32-year follow-up of the 1970 British Cohort Study. J Epidemiol Community Health.

[119] Hayley AC, Skogen JC, Øverland S, Wold B, Williams LJ, Kennedy GA (2015). Trajectories and stability of self-reported short sleep duration from adolescence to adulthood. J Sleep Res.

[120] de Melo Boff R, Liboni RP, de Azevedo Batista IP, de Souza LH, da Silva Oliveira M. Weight loss interventions for overweight and obese adolescents: a systematic review. Eat Weight Disord. 2017;22:211–29. 10.1007/s40519-016-0309-1 27542161

[121] Thivel D, Aucouturier J, Doucet É, Saunders TJ, Chaput JP. Daily energy balance in children and adolescents. Does energy expenditure predict subsequent energy intake? Appetite. 2013;60:58–64. 10.1016/j.appet.2012.09.022 23023045

[122] Beaulieu K, Hopkins M, Blundell J, Finlayson G (2016). Does habitual physical activity increase the sensitivity of the appetite control system?. A systematic review Sports Med.

[123] Lowry R, Michael S, Demissie Z, Kann L, Galuska DA. Associations of physical activity and sedentary behaviors with dietary behaviors among US high school students. J Obes. 2015;2015:876524. 10.1155/2015/876524 PMC4458549 26101666

[124] Platat C, Perrin A-E, Oujaa M, Wagner A, Haan M-C, Schlienger J-L (2006). Diet and physical activity profiles in French preadolescents. Br J Nutr.

[125] Tambalis KD, Panagiotakos DB, Psarra G, Sidossis LS. Concomitant associations between lifestyle characteristics and physical activity status in children and adolescents. J Res Health Sci. 2019;19:e00439. PMC6941623 31133628

[126] Ottevaere C, Huybrechts I, De Bourdeaudhuij I, Sjöström M, Ruiz JR, Ortega FB (2011). Comparison of the IPAQ-A and actigraph in relation to VO2max among European adolescents: the HELENA study. J Sci Med Sport.

[127] Santaliestra-Pasías AM, Dios JEL, Sprengeler O, Hebestreit A, De Henauw S, Eiben G (2018). Food and beverage intakes according to physical activity levels in European children: the IDEFICS (Identification and prevention of Dietary and lifestyle induced health EFfects In Children and infantS) study. Public Health Nutr.

[128] Saldanha-Gomes C, Marbac M, Sedki M, Cornet M, Plancoulaine S, Charles M-A (2020). Clusters of diet, physical activity, television exposure and sleep habits and their association with adiposity in preschool children: the EDEN mother-child cohort. Int J Behav Nutr Phys Act.

[129] Beaulieu K, Hopkins M, Blundell J, Finlayson G (2018). Homeostatic and non-homeostatic appetite control along the spectrum of physical activity levels: an updated perspective. Physiol Behav.

[130] Blundell JE, Gibbons C, Caudwell P, Finlayson G, Hopkins M (2015). Appetite control and energy balance: impact of exercise. Obes Rev.

[131] Craigie AM, Lake AA, Kelly SA, Adamson AJ, Mathers JC (2011). Tracking of obesity-related behaviours from childhood to adulthood: a systematic review. Maturitas.

[132] Masurier J, Mathieu ME, Fearnbach SN, Cardenoux C, Julian V, Lambert C, et al. Effect of exercise duration on subsequent appetite and energy intake in obese adolescent girls. Int J Sport Nutr Exerc Metab. 2018;1–23. 10.1123/ijsnem.2017-0352 PMC6221974 29431521

[133] Moore MS, Dodd CJ, Welsman JR, Armstrong N (2004). Short-term appetite and energy intake following imposed exercise in 9- to 10-year-old girls. Appetite.

[134] Bozinovski NC, Bellissimo N, Thomas SG, Pencharz PB, Goode RC, Anderson GH (2009). The effect of duration of exercise at the ventilation threshold on subjective appetite and short-term food intake in 9 to 14 year old boys and girls. Int J Behav Nutr Phys Act.

[135] Miguet M, Fearnbach SN, Metz L, Khammassi M, Julian V, Cardenoux C, et al. Effect of HIIT versus MICT on body composition and energy intake in dietary restrained and unrestrained adolescents with obesity. Appl Physiol Nutr Metab. 2019. 10.1139/apnm-2019-0160 31505120

[136] Thivel D, Rumbold PL, King NA, Pereira B, Blundell JE, Mathieu M-E (2016). Acute post-exercise energy and macronutrient intake in lean and obese youth: a systematic review and meta-analysis. Int J Obes (Lond).

[137] Reid RER, Thivel D, Mathieu M-E (2019). Understanding the potential contribution of a third “T” to FITT exercise prescription: the case of timing in exercise for obesity and cardiometabolic management in children. Appl Physiol Nutr Metab.

[138] Fillon A, Mathieu ME, Boirie Y, Thivel D. Appetite control and exercise: does the timing of exercise play a role? Physiol Behav. 2020;218:112733. 10.1016/j.physbeh.2019.112733 31707067

[139] Fillon A, Beaulieu K, Miguet M, Bailly M, Finlayson G, Julian V (2020). Does exercising before or after a meal affect energy balance in adolescents with obesity?. Nutr Metab Cardiovasc Dis.

[140] Mathieu M-E, Lebkowski A, Laplante E, Drapeau V, Thivel D (2018). Optimal timing of exercise for influencing energy intake in children during school lunch. Appetite.

[141] Albert M-H, Drapeau V, Mathieu M-E (2015). Timing of moderate-to-vigorous exercise and its impact on subsequent energy intake in young males. Physiol Behav.

[142] Fillon A, Mathieu ME, Masurier J, Roche J, Miguet M, Khammassi M, et al. Effect of exercise-meal timing on energy intake, appetite and food reward in adolescents with obesity: The TIMEX study. Appetite. 2020;146:104506. 10.1016/j.appet.2019.104506 31678149

